# Supplementary material for: De Novo Genome Assembly at Chromosome-Scale of Hermetia illucens (Diptera Stratiomyidae) via PacBio and Omni-C Proximity Ligation Technology
Source: Insects. 2024 Feb 17;15(2):133. doi: 10.3390/insects15020133 (PMC10889594; doi:10.3390/insects15020133)
Supplement: Supplementary file 1 [file insects-15-00133-s001.zip › insects-2868160-supplementary.pdf]

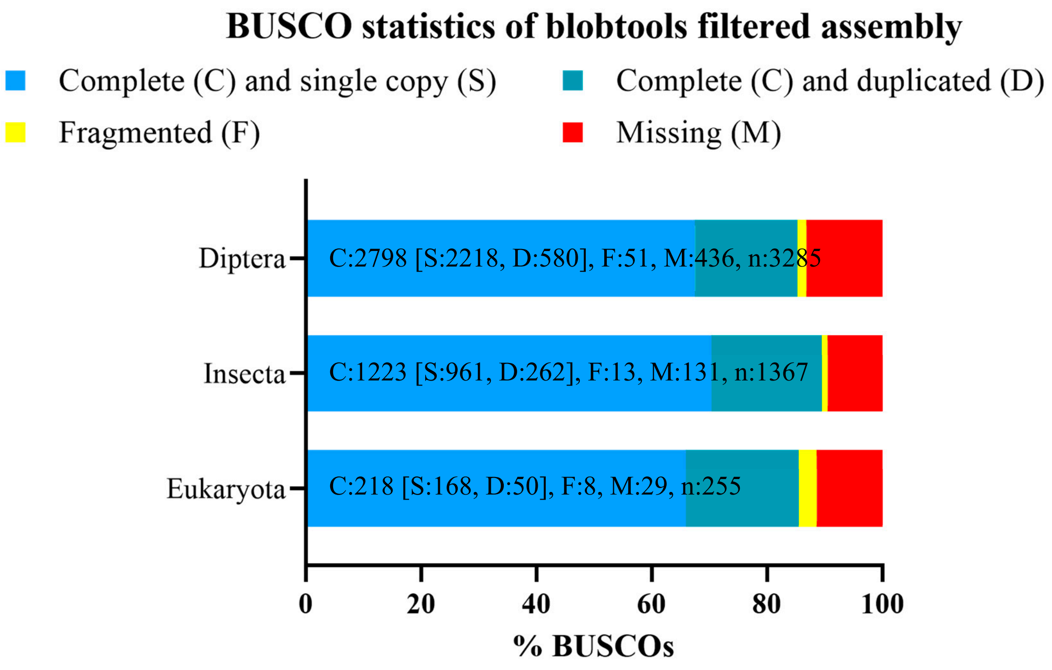

Figure S1. BUSCO scores of the blobtools filtered assembly against Diptera, Insecta and Eukaryota databases. Used databases to assess completeness against dipterans, insects and eukaryotes: “diptera_odb10”, “insecta_odb10” and “eukaryota_odb10” databases, respectively. BUSCO= Benchmarking Universal Single-Copy Orthologs.
